# The New Era of Provisional Restorative Materials: Surface Roughness Responses to Artificial Aging

**DOI:** 10.3390/polym18212728

**Published:** 2026-11-03

**Authors:** Abdulrahman Jafar Alhaddad

**Affiliations:** Oral and Maxillofacial Prosthodontics Department, Faculty of Dentistry, King Abdulaziz University, Jeddah 21589, Saudi Arabia; aalhaddad@kau.edu.sa

**Keywords:** provisional restorative materials, surface roughness, CAD/CAM, 3D printing, artificial aging, thermocycling, beverage exposure

## Abstract

Provisional restorative materials can be fabricated using conventional, subtractive, and additive manufacturing techniques; however, evidence regarding the combined effects of artificial aging and beverage exposure on the surface characteristics of these materials remains limited. This study evaluated the influence of material type, storage medium, and artificial aging on the surface roughness of provisional restorative materials fabricated using different manufacturing workflows. Ninety square-shaped specimens (15 mm × 15 mm × 1.5 mm; *n* = 10 per experimental group) were prepared from a conventional polymethyl methacrylate resin (Unifast III), a CAD/CAM material (VITA CADTemp), and a three-dimensional (3D)-printed resin (NextDent C&B MFH). All specimens were standardized using a sequential silicon carbide finishing and polishing protocol before testing. Specimens were stored in distilled water, coffee, or Coca-Cola and assessed at baseline (T0), after 15,000 thermocycles and 7 days of immersion (T1), and after 30,000 thermocycles and 14 days of immersion (T2). Surface roughness (Ra) was measured using a non-contact 3D optical profilometer. A significant interaction among material type, storage medium, and aging period was observed (*p* = 0.017). VITA CADTemp consistently exhibited the lowest surface roughness values (0.158–0.215 µm), followed by NextDent C&B MFH (0.245–0.382 µm) and Unifast III (0.337–0.617 µm), with significant differences among all materials at each evaluation period (*p* < 0.05). At T2, significant increases in surface roughness were observed in Unifast III specimens stored in coffee and Coca-Cola and in NextDent C&B MFH specimens stored in coffee (*p* < 0.05), whereas no significant changes were detected in VITA CADTemp or distilled-water groups. Within the limitations of this study, surface roughness depended on material, storage medium, and cumulative aging, with VITA CADTemp exhibiting the lowest values. Mean Ra exceeded the commonly cited 0.2 µm threshold for plaque retention in Unifast III and NextDent C&B MFH at all measurement points, and in VITA CADTemp only after coffee exposure at T2.

## 1. Introduction

Provisional fixed prostheses are used to protect prepared teeth and maintain function, esthetics, tooth position, and occlusion until delivery of the definitive prosthesis [1,2,3]. They also serve a diagnostic function, allowing assessment of the adequacy of tooth preparation and evaluation of the proposed functional, occlusal, and esthetic parameters before fabrication of the definitive prosthesis [1,4]. Accordingly, materials used for provisional restorations should maintain adequate mechanical and surface properties throughout the intended period of service [4,5].

Provisional fixed prostheses can be fabricated using conventional techniques or digital workflows based on computer-aided design and computer-aided manufacturing (CAD/CAM), including subtractive and additive manufacturing [5,6]. Additive manufacturing, commonly known as 3D printing, produces three-dimensional objects through layer-by-layer fabrication based on a digital design [7]. Compared with subtractive manufacturing, 3D printing enables the fabrication of complex geometries with reduced material waste and fewer limitations related to milling techniques [8].

Surface roughness of provisional fixed prostheses is clinically important because increased surface irregularities may favor microbial plaque retention and biofilm development [9]. The surface roughness and plaque accumulation of provisional restorative materials may vary according to their material composition and manufacturing workflow [9,10]. Furthermore, the surface characteristics of 3D-printed resins are influenced by material composition and processing parameters. Previous studies have shown that resin type, printing layer thickness, build orientation, and post-processing procedures can affect the surface roughness of 3D-printed dental resins [8,11,12,13]. These findings emphasize the importance of evaluating the surface roughness of 3D-printed provisional fixed prostheses.

Although provisional restorations are generally intended for temporary use, they may be required for prolonged periods during complex treatment procedures [14]. The surface properties of provisional restorative materials can be affected by conditions encountered during clinical service [15]. During this period, provisional restorative materials are exposed to beverages with different compositions and pH values [16]. Exposure to acidic media has been associated with changes in the surface roughness of resin-based restorative materials [16,17]. Coffee, Coca-Cola, and distilled water were selected to represent storage media with different characteristics. Coffee and cola-based beverages are widely consumed and commonly investigated in dental-material studies. Coffee contains chromogenic compounds that may interact with polymeric materials, while the acidic pH of Coca-Cola may contribute to surface degradation. Distilled water served as a control medium to assess changes without the additional staining or acidic components present in the beverages [16,18,19].

Thermocycling is commonly used as an artificial aging method in which dental materials are exposed to temperature fluctuations in a moist environment to simulate thermal conditions in the oral cavity [20]. Previous studies have reported inconsistent effects of artificial aging on the surface roughness of provisional restorative materials, with significant changes observed in some materials, whereas others remained unaffected [5,21,22].

Although previous studies have evaluated the effects of thermocycling and beverage exposure on digitally fabricated provisional materials and other resin-based restorative materials, limited evidence is available comparing conventionally fabricated, subtractively manufactured, and additively manufactured provisional restorative materials under identical aging conditions and sequential thermocycling regimens [15,21,22]. Therefore, this study aimed to evaluate the individual and interactive effects of material type, storage medium, and cumulative artificial aging on the surface roughness of provisional restorative materials fabricated using conventional, subtractive, and additive techniques. The null hypothesis was that material type, storage medium, and artificial aging, as well as their interactions, would have no significant effect on the surface roughness of the tested provisional restorative materials.

## 2. Materials and Methods

### 2.1. Materials and Sample Size

This in vitro study evaluated the effect of artificial aging on the surface roughness of three commercially available materials indicated for provisional fixed prostheses and fabricated using different techniques. Surface roughness was evaluated at three time points: at baseline, after 15,000 thermocycles followed by 7 days of storage in the assigned medium, and after an additional 15,000 thermocycles followed by continued storage in the assigned medium until day 14.

The investigated materials were a conventionally polymerized resin (Unifast III; GC Corp., Tokyo, Japan), a prefabricated CAD/CAM material for subtractive manufacturing (VITA CADTemp; VITA Zahnfabrik, Bad Säckingen, Germany), and a photopolymerizable resin for additive manufacturing (NextDent C&B MFH; 3D Systems, Soesterberg, The Netherlands). The composition and manufacturer information of the investigated materials are presented in Table 1.

A priori power analysis was performed using G*Power (version 3.1; Heinrich Heine University Düsseldorf, Düsseldorf, Germany) for a repeated-measures ANOVA with a within–between interaction. Assuming a medium effect size (f = 0.25) according to Cohen’s recommendations for behavioral and biomedical research, a significance level of 0.05, and a power of 95%, the minimum required sample size was 90 specimens (*n* = 10 per experimental group).

### 2.2. Specimen Preparation

Square-shaped specimens (15 mm × 15 mm × 1.5 mm) were fabricated from each material. The dimensions of all specimens were verified after finishing and polishing using a digital caliper (Mitutoyo Corp., Kawasaki, Japan). All specimens met the specified dimensions after finishing and polishing.

#### 2.2.1. Additively Manufactured Specimens

For the additively manufactured specimens, an STL file was generated using Tinkercad, an online 3D modeling platform. To compensate for material removal during finishing and polishing procedures, the specimens were designed with a 0.5 mm oversize allowance for finishing and polishing. The STL file was imported into Asiga Composer software (version 1.1.7; Asiga, Alexandria, Australia), oriented at 90° to the build platform, and automatically provided with support structures. The supports were attached to the specimen edge facing the build platform, away from the test surface to prevent support removal from altering surface roughness measurements. A vertical printing orientation was selected to standardize specimen fabrication and maintain methodological consistency with previously published studies evaluating 3D-printed provisional restorative materials [8,12]. The specimens were fabricated from NextDent C&B MFH resin using a DLP 3D printer operating at a wavelength of 385 nm (Asiga MAX UV; Asiga, Alexandria, Australia) at a layer thickness of 50 μm, using the official manufacturer-validated print profile.

After printing, the specimens were removed from the build platform, and the support structures were detached. The specimens were ultrasonically cleaned in >99% isopropyl alcohol for 3 min (Power Sonic 405; Whashin Co., Seoul, Korea) and air-dried for 15 min. The specimens were then post-polymerized for 30 min in a NextDent LC-3DPrint Box (3D Systems, Soesterberg, The Netherlands), according to the manufacturer’s recommendations (NextDent C&B MFH instructions for use; IBNCM202001_1, 10 March 2020).

#### 2.2.2. Subtractively Manufactured Specimens

For the subtractively manufactured specimens, VITA CAD-Temp multiColor blocks (CTM-40; 15.5 mm × 19 mm × 39 mm) were sectioned into 2-mm-thick square-shaped specimens using a precision saw (TechCut 5; Allied, CA, USA) equipped with a 5-inch diamond blade under continuous water cooling. Sectioning was performed at 3000 rpm under low-load conditions and a feed rate of 0.8 mm/min.

#### 2.2.3. Conventionally Fabricated Specimens

For the conventionally fabricated specimens, UNIFAST III powder and liquid were mixed at the manufacturer-recommended ratio of 1 g of powder to 0.5 mL of liquid. The components were manually mixed with a plastic spatula for 10–15 s and immediately poured into a custom-made resin mold (15 mm × 15 mm × 2 mm in dimensions) coated with a thin layer of separating medium (DFS ISOFIX; DFS, Riedenburg, Germany). The mold was positioned between two glass slabs, and finger pressure was applied during polymerization. The specimens were then polymerized in a pressure pot (S-U-Acrylmat; Schuler-Dental, Ulm, Germany) at 2 bar for 10 min.

After polymerization, the specimens were removed from the mold and excess material was removed using a carbide bur (H251EF.11.060; Brasseler, Savannah, GA, USA) at 30,000 rpm.

#### 2.2.4. Finishing and Polishing

Before finishing and polishing, all specimens were visually inspected under ambient illumination for visible voids, porosity, cracks, and edge irregularities. Four specimens with visible defects (two NextDent C&B MFH and two Unifast III) were excluded and replaced. No VITA CADTemp specimens were excluded.

All specimens were finished and polished using a grinding-polishing machine (EcoMet 250; Buehler, Lake Bluff, IL, USA) to standardize the specimen dimensions and surface characteristics. The specimens were sequentially finished with silicon carbide abrasive papers (P600, P800, and P1200; Buehler, Lake Bluff, IL, USA) under continuous water cooling at 300 rpm for a total of 3 min across the three grit sizes. The specimens were then polished with P2500 abrasive paper under continuous water cooling for an additional 1 min. Finishing and polishing were performed by a single operator under manually applied pressure without a predefined load. The operator was blinded to the experimental group allocation throughout the study to minimize variability in specimen preparation. Silicon carbide abrasive papers were replaced after completion of each experimental subgroup (*n* = 10) to minimize the effects of abrasive wear and maintain consistent polishing efficiency throughout specimen preparation. After polishing, the specimens were ultrasonically cleaned for 10 min, rinsed with water, and dried. This protocol was selected to standardize surface preparation across the tested materials and obtain flat surfaces suitable for profilometric assessment, consistent with the general approach used in previous comparative research on provisional restorative materials [9].

### 2.3. Artificial Aging

The specimens from each material were randomly assigned to three storage-medium subgroups (*n* = 10) using a computer-generated randomization sequence in Microsoft Excel (Microsoft Corp., Redmond, WA, USA): coffee, Coca-Cola, or distilled water (Table 2). The coffee solution was prepared by dissolving 2.8 g of instant coffee (Nescafé Classic) in 150 mL of boiling distilled water, following a previously published methodology [16], and was subsequently maintained at 37 °C during specimen storage. For the Coca-Cola group, 150 mL of the beverage was used without further preparation. The pH values of the beverages were obtained from previous studies [18,19,23] and subsequently verified using a Flintronic Digital pH Meter (Shenzhen Bamale E-Commerce Co., Ltd., Shenzhen, China). Before pH measurement, the meter was calibrated using pH 4.00 and 6.86 buffer solutions, each prepared by dissolving the supplied powder separately in distilled water. The pH measurements were performed at room temperature (≈25 °C). The pH of the storage media was measured at the beginning of the experiment. Although the media were renewed daily, changes in pH between replacements were not monitored.

Baseline (T0) measurements were obtained after the specimens had been stored in distilled water at 37 °C for 24 h to standardize the initial storage conditions.

After baseline measurements, all specimens were subjected to 15,000 thermocycles between water baths maintained at 5 °C and 55 °C using a thermocycling unit (SD Mechatronik Thermocycler; SD Mechatronik, Westerham, Germany), with a dwell time of 30 s in each bath and a transfer time of 10 s. The initial interval of 15,000 thermocycles was selected to divide the cumulative 30,000-cycle exposure into two equal stages, allowing surface roughness to be assessed after each stage. The thermocycling protocol was independently selected for this comparative laboratory assessment and did not follow a specific ISO standard. These intervals were intended for comparative assessment, without assigning a direct equivalence to clinical service duration. The specimens were then immersed in their assigned storage media in dark containers and maintained at 37 °C in an incubator (General Purpose Incubator 6T2-2; Sheldon, OR, USA) throughout the storage period. The storage media were renewed daily. Surface roughness was then measured after 15,000 thermocycles and 7 days of storage (T1).

Following the T1 measurements, the specimens were subjected to an additional 15,000 thermocycles under the same conditions and then returned to their respective storage media for a further 7 days, with daily renewal of the media. The final surface roughness measurement was obtained after a total of 30,000 thermocycles and 14 days of storage (T2).

### 2.4. Surface Roughness Measurement

Surface roughness (Ra, µm) was measured using a non-contact 3D optical profilometer (Contour GT-K 3D; Bruker, Billerica, MA, USA) using vertical scanning interferometry. Before data acquisition, the accuracy of the profilometer was verified using a step-height calibration standard of known nominal height in the 1× vertical scanning interferometry mode, following the manufacturer’s instructions. Measurements were performed using a 5× Michelson objective with a 2 × 2 mm field of view. The scan speed was set to 1×, with a 4% modulation threshold for accepting valid measurement points. A Gaussian regression filter was applied, with a cut-off distance of 0.08 mm. Surface topography data were acquired and analyzed using Vision64 software (version 5.41, update 4; Bruker, Billerica, MA, USA). Three different regions of each specimen were scanned. Within each region, Ra was calculated from six linear profiles: three parallel profiles in the horizontal direction and three in the vertical direction. The six Ra values were averaged to obtain one reading per region, and the readings from the three regions were then averaged to determine the specimen’s mean surface roughness (Ra, µm). The same three measurement regions were relocated at T0, T1, and T2 using a custom specimen holder and reference markings. All measurements were performed by a single calibrated operator blinded to both material type and storage-medium allocation.

### 2.5. Statistical Analysis

Data were analyzed using SPSS statistical software (IBM SPSS Statistics, version 31.0.1.0; IBM Corp., Armonk, NY, USA). Data normality was assessed using the Shapiro–Wilk test, and homogeneity of variances was evaluated using Levene’s test. Mean surface roughness values and standard deviations (SD) were obtained. A three-way mixed-design ANOVA with repeated measures was used to evaluate the effects of material type, storage medium, and measurement time point (T0, T1, and T2), as well as their interactions, on surface roughness. Sphericity was assessed using Mauchly’s test for the measurement time point effect and its interactions with material type and storage medium (W = 0.996, *p* = 0.860); no sphericity correction was required. Pairwise comparisons were performed with Bonferroni adjustment for multiple comparisons. Comparisons among storage media within each material and measurement time point were performed using two-sided Welch’s independent-samples *t*-tests with Bonferroni adjustment. The significance level was set at α = 0.05.

## 3. Results

The three-way mixed-design ANOVA (Table 3) revealed a significant material × storage medium × measurement time point interaction (*F*(8, 162) = 2.43, *p* = 0.017; partial η^2^ = 0.107). Significant material × measurement time point, storage medium × measurement time point, and material × storage medium interactions were also detected (*p* < 0.001). The main effects of material, storage medium, and measurement time point were significant (*p* < 0.001). Material had the largest reported partial η^2^ (0.908).

Surface roughness mean values of the investigated materials at baseline and after aging are summarized in Table 4. At each measurement point, VCTemp exhibited the lowest overall Ra values, ND showed intermediate values, and UF exhibited the highest values. Pairwise comparisons demonstrated significant differences among all three materials within each storage medium and measurement time point (*p* < 0.05).

Within each material-storage medium combination, comparisons across measurement time points showed that; Ra increased significantly in UF specimens assigned to the coffee from T0 to T1 (mean difference, 0.076 µm; 95% CI, 0.025 to 0.127; *p* = 0.002), from T0 to T2 (mean difference, 0.219 µm; 95% CI, 0.166 to 0.272; *p* < 0.001), and from T1 to T2 (mean difference, 0.143 µm; 95% CI, 0.089 to 0.197; *p* < 0.001). For UF specimens stored in Coca-Cola, Ra increased significantly from T0 to T2 (mean difference, 0.188 µm; 95% CI, 0.135 to 0.241; *p* < 0.001) and from T1 to T2 (mean difference, 0.140 µm; 95% CI, 0.086 to 0.194; *p* < 0.001). In ND specimens stored in coffee, Ra increased significantly from T0 to T2 (mean difference, 0.112 µm; 95% CI, 0.059 to 0.165; *p* < 0.001) and from T1 to T2 (mean difference, 0.081 µm; 95% CI, 0.027 to 0.135; *p* = 0.001). In contrast, the increases observed in VCTemp specimens stored in coffee were not statistically significant from T0 to T2 (mean difference, 0.050 µm; 95% CI, −0.003 to 0.103; *p* = 0.071) or from T1 to T2 (mean difference, 0.040 µm; 95% CI, −0.014 to 0.094; *p* = 0.222). No statistically significant changes were detected across the three measurement time points in the distilled-water group. At T1, UF specimens assigned to the coffee group exhibited significantly higher Ra values than those assigned to the distilled-water and Coca-Cola groups (*p* < 0.05), and no significant differences among the storage media were observed for VCTemp or ND.

At T2, after 30,000 thermocycles and 14 days of immersion in the assigned storage media, UF specimens in the coffee and Coca-Cola groups exhibited significantly higher Ra values than those in the distilled-water group (*p* < 0.001, *p* = 0.01, respectively), with no significant difference between the coffee and Coca-Cola. For VCTemp, the coffee group exhibited significantly higher Ra values than the distilled-water group (*p* = 0.02), whereas the Coca-Cola group showed intermediate values, with no statistically significant pairwise differences relative to either group. For ND, significant differences were identified among all three storage media (*p* < 0.05); the highest Ra values were recorded in the coffee group, followed by the Coca-Cola and distilled-water groups.

## 4. Discussion

This study investigated the individual and interactive effects of material type, storage medium, and cumulative artificial aging on the surface roughness of provisional restorative materials fabricated using conventional, subtractive, and additive manufacturing. The artificial aging protocol comprised sequential thermocycling and immersion in the assigned storage media and was evaluated at three measurement time points. The significant material × storage medium × measurement time point interaction demonstrated that changes in Ra were dependent on the combined influence of the tested material, immersion medium, and extent of aging, highlighting the importance of assessing these factors simultaneously rather than in isolation. Therefore, the null hypothesis was rejected. This finding emphasizes the multifactorial nature of surface degradation in provisional restorative materials and supports the value of evaluating cumulative aging protocols that incorporate both thermocycling and beverage exposure. Furthermore, among all investigated factors, material type demonstrated the largest effect size (partial η^2^ = 0.908), indicating that differences among the investigated materials exerted a substantially greater influence on surface roughness than storage medium or aging duration. This finding highlights the importance of material selection when long-term provisionalization is anticipated.

Significant differences in Ra were observed among the three tested materials. From a clinical perspective, these findings suggest that the surface quality of provisional restorations may vary according to the selected material when prolonged service is anticipated. VCTemp consistently exhibited the lowest Ra values, ND showed intermediate values, and UF demonstrated the highest surface roughness throughout the study. This finding is consistent with previous studies reporting differences in surface roughness among provisional restorative materials fabricated using different production methods [9,21]. The comparatively low Ra values of VCTemp may be attributed to the industrial polymerization of CAD/CAM blocks under controlled conditions, which has been reported to produce a more homogeneous polymer network with a high degree of conversion and reduced porosity [5,21]. ND is a photopolymerized, dimethacrylate-based 3D-printed resin with a lower reported inorganic filler content than VCTemp (Table 1). Its intermediate Ra values may be associated with its resin formulation, as the surface roughness of printed provisional resins is influenced by material composition [12,22]. Moreover, solvent cleaning and post-polymerization conditions can influence the surface properties and degree of conversion of 3D-printed resins [13]. In contrast, UF is a self-cured PMMA resin prepared by manual powder-liquid mixing. Its higher Ra values may be related to air entrapment and void formation during manual mixing [5,9], as well as surface irregularities associated with polymerization under less controlled conditions [21]. However, these proposed explanations were not directly investigated in the present study.

The effect of artificial aging on Ra varied according to the tested material and storage medium. Coffee produced the highest mean Ra values at T2 for all three materials; however, significant temporal increases in the coffee groups were detected only for UF and ND. In the UF coffee group, Ra increased significantly from T0 to T1 and again from T1 to T2, whereas in the ND coffee group, a significant increase was detected at T2 relative to both T0 and T1. UF specimens stored in Coca-Cola also exhibited significantly higher Ra at T2 than at T0 and T1. In contrast, no significant temporal changes were observed in any VCTemp or distilled-water group. The absence of significant changes in Ra in the distilled-water groups is consistent with a previous study that reported no change in the surface roughness of provisional materials after 14 days of immersion in distilled water [16].

Although coffee yielded the highest mean Ra values for all tested materials at T2, coffee produced a significantly higher Ra than Coca-Cola only in ND, indicating that its relative effect varied among the tested materials. This variation in response is consistent with previous evidence showing that beverage-related changes in surface roughness depend on the restorative material, immersion medium [16,24,25], exposure duration [26], and aging protocol [27,28]. Previous studies reported a similar pattern, with coffee immersion resulting in significantly greater surface roughness changes than immersion in distilled water or cola in direct resin composites and PEEK-based materials [25,29]. Acidic conditions may contribute to softening and surface degradation of resin composites [18]. However, this pattern cannot be attributed solely to acidity, because the coffee solution was less acidic than Coca-Cola (pH 4.86 versus 2.69). The chemical composition and polarity of the solution may also influence polymer solubility and subsequent surface degradation [24]. In addition, the ionic composition of the storage medium may influence ion exchange at filler surfaces and the leaching of filler constituents from resin composites [30]. This mechanism may be relevant to the filler-containing VCTemp and ND materials, but it would not explain the larger changes observed in the essentially unfilled UF. However, the ionic composition of the media and filler-element release were not measured in this study. Conversely, coffee thermocycling was reported to have little effect on the post-polishing Ra of additively and subtractively manufactured definitive resin materials [28]. This discrepancy may reflect differences in material composition, surface treatment, the sequence of aging procedures, and the number of thermal cycles applied.

The effects of the storage media became more evident with cumulative aging, as significant medium-related differences were limited to UF at T1 but were detected in all tested materials at T2. This pattern suggests that the influence of the storage media varied according to the tested material and became more apparent after cumulative aging. Both the immersion duration and the number of thermal cycles increased between T1 and T2; therefore, their individual contributions cannot be isolated. Thermocycling was performed in water. Additional control groups subjected to immersion without thermocycling or thermocycling without subsequent immersion in the assigned media would have been needed to distinguish the contributions of these aging procedures. Nevertheless, thermocycling may enhance the effects of prolonged immersion in the storage media by inducing repeated thermal contraction and expansion and facilitating water diffusion into the polymer networks [20,21,22,31]. This may consequently promote matrix plasticization and hydrolytic degradation and generate internal or interfacial stresses, thereby compromising surface integrity and increasing Ra [21,31]. The material-dependent response is consistent with evidence that thermocycling increased Ra in self-cured PMMA and an SLA-printed resin, while producing no significant change in a light-cured UDMA-based material [32].

Clinically, these findings suggest that the anticipated duration of provisionalization and the patient’s dietary habits may be relevant when selecting materials for provisional fixed prostheses. A surface roughness of 0.2 µm is commonly cited as a threshold for bacterial plaque retention [33]. Although ND exhibited significantly lower Ra than UF, both materials exceeded the 0.2 µm threshold at baseline. VCTemp exceeded this threshold only after coffee exposure at T2 (0.215 µm). Nevertheless, the clinical significance of these observations should be interpreted cautiously, as plaque accumulation was not assessed in the present study and is influenced by multiple factors other than surface roughness.

Nonetheless, this study has several limitations. Only one commercial material was selected to represent each manufacturing workflow; therefore, the observed differences should be interpreted as material-specific findings rather than definitive evidence of the superiority of a particular fabrication technique. Furthermore, the use of flat, uniformly polished specimens did not reproduce the anatomical contours, marginal geometry, or surface-finishing procedures of clinical provisional fixed prostheses. Moreover, the standardized silicon carbide grinding and polishing procedure removed the original surface characteristics associated with each fabrication method; therefore, the findings should be interpreted as reflecting the behavior of standardized polished surfaces rather than the native surfaces produced directly by conventional, subtractive, or additive manufacturing techniques. Variations in manually applied pressure and potential abrasive wear within each subgroup during finishing and polishing may have contributed to some variability in surface preparation. The aging protocol, which combined continuous immersion with sequential thermocycling, also did not fully simulate intermittent beverage consumption or intraoral conditions such as salivary dilution, acquired pellicle formation, oral hygiene procedures, and mechanical loading. Furthermore, thermocycling in water exposed all specimens to additional water beyond storage in their assigned media. The individual contributions of water exposure and thermal cycling could therefore not be distinguished. In addition, only Ra was evaluated; therefore, the proposed mechanisms of surface degradation were not directly confirmed by microscopic or chemical analyses. Future studies should incorporate three-dimensional surface characterization, chemical analysis, wear simulation, and biofilm assessment under more clinically representative aging conditions.

## 5. Conclusions

Within the limitations of this in vitro study, surface roughness was influenced by the interactive effects of material type, storage medium, and cumulative artificial aging. VITA CADTemp consistently demonstrated the lowest surface roughness values, followed by NextDent C&B MFH and Unifast III. Prolonged aging increased surface roughness primarily in coffee-exposed specimens and in Unifast III specimens exposed to Coca-Cola, whereas specimens stored in distilled water remained relatively stable. Mean Ra values for Unifast III and NextDent C&B MFH exceeded the commonly cited 0.2 µm threshold for plaque retention at all measurement points. VITA CADTemp remained below this threshold in all groups except after 30,000 thermocycles and 14 days of immersion in coffee (0.215 µm).

## Figures and Tables

**Table 1:** Provisional restorative materials investigated in the study.

Fabrication Technique	Material	Composition	Manufacturer
Conventional	Unifast III (UF)	**Powder components:** polymethyl methacrylate (PMMA), copolymer of ethyl methacrylate and methyl methacrylate, barbituric acid derivative, organic copper compound, and pigment. **Liquid components:** methyl methacrylate (90–<100%), N,N-dimethyl-p-toluidine (2.5–<5%), ethylene glycol dimethacrylate (1–<2.5%), propylidynetrimethyl trimethacrylate (1–<2.5%). **Other constituents: UV absorber:** 2-(2H-benzotriazol-2-yl)-p-cresol (≥0.25–<0.5%); inhibitor: butylated hydroxytoluene (0.1–<0.2%); titanium dioxide (powder, 0.5–<1%).	GC Corporation
Subtractive manufacturing (Milling)	VITA CADTemp (VCTemp)	**Organic matrix:** polymethyl methacrylate (PMMA) (82–86%) **Inorganic component:** silicon dioxide (SiO_2_) (14–17%); **pigments:** <1%	VITA Zahnfabrik
Additive manufacturing (3D printing)	NextDent C&B MFH (ND)	**Organic matrix:** 7,7,9(or 7,9,9)-trimethyl-4,13-dioxo-3,14-dioxa-5,12-diazahexadecane-1,16-diyl bismethacrylate (50–75%), HEMA (<25%), ethoxylated bisphenol A dimethacrylate (<10%), ethylene dimethacrylate (<10%) **Inorganic components:** SiO_2_ (1–5%), TiO_2_ (<0.1%); **photoinitiator:** diphenyl(2,4,6-trimethylbenzoyl)phosphine oxide (1–5%); **inhibitor:** mequinol (<0.1%).	3D Systems

**Table 2:** Storage media used in this study.

Storage Media	Manufacturer	pH [18,19,23]	Measured pH
Coffee	Nescafé, Nestlé UK Ltd. (York, UK)	4.9	4.86
Coca Cola	Coca-Cola Co. (Atlanta, GA, USA)	2.6	2.69
Distilled water	Nuve water distiller ND8 (Ankara, Turkey)	6.9	6.93

**Table 3:** Three-way mixed-design ANOVA evaluating the effects of material, storage medium and aging time on surface roughness of the investigated materials.

Source of Variation	df	F	*p* Value	Partial η^2^
**Material**	2, 81	400.199	<0.001	0.908
**Media**	2, 81	30.998	<0.001	0.434
**Material × Media**	4, 81	5.982	<0.001	0.228
**Time**	2, 162	58.704	<0.001	0.420
**Material × Time**	4, 162	12.492	<0.001	0.236
**Media × Time**	4, 162	10.998	<0.001	0.214
**Material × Media × Time**	8, 162	2.430	0.017	0.107

**Table 4:** Mean surface roughness of the provisional restorative materials according to storage medium and measurement time.

Material	Media	Surface Roughness (Ra) (µm) Mean (± SD)		
		Baseline (T0)	T1	T2
**Unifast III (UF)**	Distilled water	0.362 ± 0.05 ^Aa^	0.368 ± 0.04 ^Aa^	0.388 ± 0.05 ^Aa^
	Coffee	0.398 ± 0.07 ^Aa^	0.474 ± 0.09 ^Bb^	0.617 ± 0.05 ^Cb^
	Coca-Cola	0.337 ± 0.08 ^Aa^	0.385 ± 0.05 ^Aa^	0.525 ± 0.11 ^Bb^
**VITA CADTemp (VCTemp)**	Distilled water	0.158 ± 0.02 ^Aa^	0.166 ± 0.02 ^Aa^	0.169 ± 0.02 ^Aa^
	Coffee	0.165 ± 0.02 ^Aa^	0.175 ± 0.04 ^Aa^	0.215 ± 0.04 ^Ab^
	Coca-Cola	0.168 ± 0.01 ^Aa^	0.170 ± 0.02 ^Aa^	0.191 ± 0.03 ^Aab^
**NextDent C&B MFH (ND)**	Distilled water	0.245 ± 0.04 ^Aa^	0.253 ± 0.04 ^Aa^	0.254 ± 0.05 ^Aa^
	Coffee	0.270 ± 0.03 ^Aa^	0.301 ± 0.06 ^Aa^	0.382 ± 0.06 ^Bc^
	Coca-Cola	0.270 ± 0.07 ^Aa^	0.283 ± 0.07 ^Aa^	0.315 ± 0.04 ^Ab^
In each row, different uppercase superscript letters indicate significant differences between different measurement times T0, T1 and T2 within each material and each beverage (*p* < 0.05). In each column, different lowercase superscript letters indicate significant differences between different beverages within the same material and measurement time T0, T1 and T2 (*p* < 0.05). Within each storage medium and measurement time point, pairwise comparisons showed significant differences among all three materials (*p* < 0.05).				

## Data Availability

The original contributions presented in the study are included in the article; further inquiries can be directed to the corresponding author.
